# Prehospital assessment of patients with abdominal pain triaged to self-care at home: an observation study

**DOI:** 10.1186/s12873-022-00649-x

**Published:** 2022-06-03

**Authors:** Glenn Larsson, Peter Hansson, Emelie Olsson, Johan Herlitz, Magnus Andersson Hagiwara

**Affiliations:** 1 Centre for Prehospital Research, Faculty of Caring Science, Work Life and Social Welfare, University of Borås, SE-501 90 Borås, Sweden; 2 Department of Prehospital Emergency Care, Sahlgrenska University Hospital, Gothenburg, Sweden; 3 NU Hospital Group (NU), Department of Ambulance Care, SE- 461 85, Trollhättan, Sweden

**Keywords:** Prehospital, EMS, Assessment, Triage, Self-care

## Abstract

**Background:**

Patients who call for emergency medical services (EMS) due to abdominal pain suffer from a broad spectrum of diseases, some of which are time sensitive. As a result of the introduction of the concept of ‘optimal level of care‘, some patients with abdominal pain are triaged to other levels of care than in an emergency department (ED). We hypothesised that it could be challenging in a patient safety perspective.

**Aim:**

This study aims to describe consecutive patients who call for EMS due to abdominal pain and are triaged to self-care by EMS clinicians.

**Methods:**

This was an observational study performed in an EMS organisation in Western Sweden during 2020. The triage tool Rapid Emergency Triage and Treatment System (RETTS), which included Emergency Signs and Symptom (ESS) codes, was used to find medical records where patients with abdominal pain have been triaged to self-care and 194 patients was included in the study.

**Results:**

Of total 48,311 ambulance missions, A total of 1747 patients were labelled with ESS code six (abdominal pain), including 223 (12.8%) who were given the code for self-care and 194 who were further assessed by the research group. Of these patients, 32 (16.3%) had a return visit within 96 hours due to the same symptoms and 11 (5.6%) were hospitalised. In six of these patients, the EMS triage was evaluated retrospectively and assessed as inappropriate. These patients had a final diagnosis of ruptured abdominal aneurysm (*n* = 1), acute appendicitis with peritonitis (*n* = 2) and acute pancreatitis (*n* = 3). All these patients required extensive evaluation and different treatments, including acute surgery, antibiotics and fluid therapy.

**Conclusion:**

Amongst the 1747 patients assessed by EMS due to abdominal pain, 223 (12.8%) were triaged to self-care. Of the 194 patients who were further assessed, 16.3% required a return visit to the ED within 96 hours and 5.6% were hospitalised. Six patients had obvious time-sensitive conditions. Our study highlights the difficulties in the early assessment of abdominal pain and the requirement for an accurate decision support tool.

## Background

An increasing demand for emergency medical services (EMS) has been observed both nationally and internationally [1]. At the same time, a large proportion of EMS patients who have been assessed do not require further EMS interventions [2, 3]. There are several reasons why an increasing number of patients seek EMS help without an actual need for emergency care. Among others, growing health awareness, poor accessibility to primary care services, population growth, ageing and socio-demographic factors have been suggested as some of these reasons [4]. All these factors have changed EMS working routines. Instead of transporting most patients to the emergency department (ED), the concept of ‘the optimal level of care’ has been introduced. This means that EMS teams assess whether a patient can stay at home with self-care instructions, be referred to primary care, be transported to the ED or be transferred directly to specialist examination or treatment [3]. This places great demands on an EMS team’s ability to assess and triage a patient safely to alternative levels of care. A Finnish study revealed that triage to the optimal level of care is a patient-safe method used in EMS units with few adverse events and a very low rate of death related to self-care decisions [5]. EDs can also benefit from patient safety if EMS transport fewer patients to EDs, given that overcrowding is a known ED patient safety threat [6].

However, some symptoms can be connected to higher patient safety risks. For example, neurological symptoms, such as dizziness, are difficult to distinguish from stroke [7, 8]. Sepsis is another condition wherein prehospital identification is quite challenging [9]. Furthermore, chest pain is a symptom wherein the majority of EMS patients have a low-risk condition without the medical need for acute hospital treatment, although 16% have time-sensitive conditions in need of rapid transport to hospital care facilities [10].

When it comes to the concept of ‘the optimal level of care’, abdominal pain is another symptom with potential patient safety risks and hundreds of differential diagnoses, including some time-sensitive diagnoses [11]. Furthermore, abdominal pain is a top five dispatch symptom [12] and the second largest amongst patients triaged to self-care or primary care by EMS [2]. Compared to EDs, EMS have limited methods to distinguish non-urgent reasons for abdominal pain from those requiring urgent interventions in hospitals.

To the best of our knowledge, no previous study has examined the safety of prehospital patients with abdominal pain triaged to self-care by EMS. We hypothesised that it is challenging to safely assess patients in the prehospital setting and to triage them to self care. Therefor the aims of our study were to:

1. investigate requirement of hospitalisation and incidence of incorrect triage by mapping return visits within 4 days with the same symptoms amongst patients with abdominal pain who are triaged to self-care; and
2. investigate which examinations are performed on site by EMS.

## Methods

### Study design

EMS and hospital medical records were examined in this retrospective observational study. The model for processes in chart review studies suggested by Kaji et al. [13] was used as a guide for the study’s design and methodology. The study proposes 10 important steps to avoid bias in chart review studies as for example systematic data collection and abstractor training.

### Population and setting

The study was conducted in an EMS organisation in Western Sweden consisting of six ambulance stations serviced by 27 ambulance units. It has approximately 55,000 EMS missions annually. An ambulance is staffed by at least one registered nurse (RN) with or without 1 year of specialist education in prehospital care. The other crew member can also be an RN or an emergency medical technician (EMT) with an assistant nurse education and a one-year EMT education [14].

According to the local guidelines followed by the EMS organisation in this study, the ambulance team should triage a patient to the optimal level of care. First, all patients are assessed on site at home and then triaged to the optimal level of care (e.g. self-care at home, primary care or ED). The RN, who is responsible for the triage, refers to the clinical guidelines, a triage tool, a triage handbook and telephone contact with ED physicians.

**Fig. 1:**
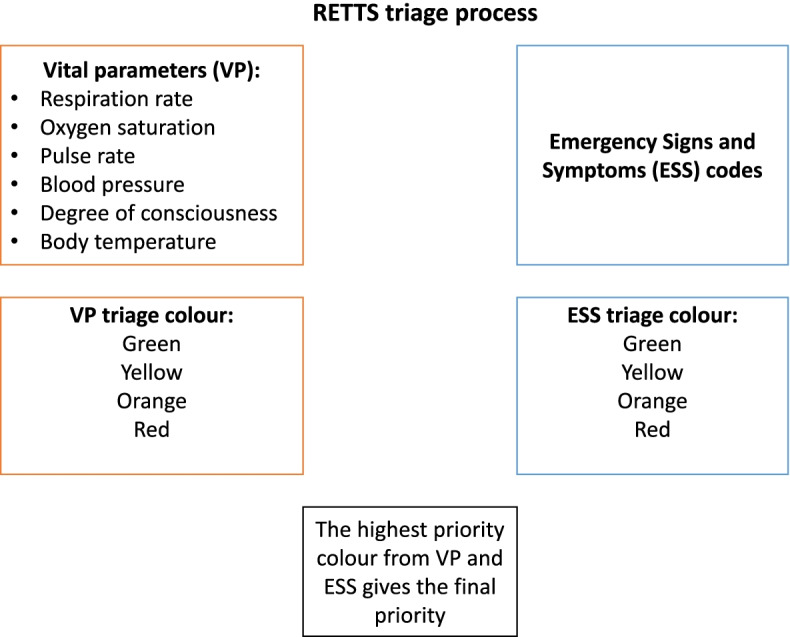
The RETTS triage process

The EMS organisation is using the Rapid Emergency Triage and Treatment System (RETTS) [15] as a triage tool. The triage tool assigns each patient a triage colour that defines the priority designation related to waiting time for a doctor’s assessment at the hospital. The red triage colour stands for ‘life-threatening’, orange for ‘potentially life-threatening’, yellow for ‘non-life-threatening’, green for ‘non-life-threatening and not in need of immediate care’ and blue for ‘no need for triage’. Triage colour is based on two variables: vital signs and type of symptoms. The type of symptoms can be divided according to different Emergency Signs and Symptoms (ESS) codes (Fig. 1). Within this scheme, the ESS code for abdominal pain is ‘6’. Thus, RETTS and EES codes was described in this study in order to give information on how patients were prioritised and how reason for contact with EMS was assessed.

The organisation also uses a symptom-based web decision support system (triage handbook). By clicking on a symptom, the EMS team can access suggestions of examination, diagnoses and advice for further processing. Except as described in guidelines, specific training of assessment in connection with abdominal pain has not been carried out in the included organisation.

The included EMS organisation has the following mandatory examinations and actions in connection to triage to self-care amongst patients with abdominal pain:

- First survey with the assessment of airway, breathing, circulation and disability (ABCD)
- Second survey with anamnesis according to Signs and symptoms, Allergies, Medications, Pertinent past history, Last oral intake, Events leading to the injury or illness, Onset, Provokes or Palliates, Quality, Radiates, Severity and Time [16]
- Measurement of vital signs, including respiration rate, oxygen saturation, pulse rate, blood pressure, degree of consciousness and body temperature
- Focused assessment in connection to abdominal pain, including inspection, palpation and auscultation of abdomen and blood test of serum glucose
- RETTS colour blue, green or yellow
- Contact with a physician
- Use of triage handbook with descriptions of self-care advice
- Written information to the patient

## Inclusion and exclusion criteria

**Fig. 2:**
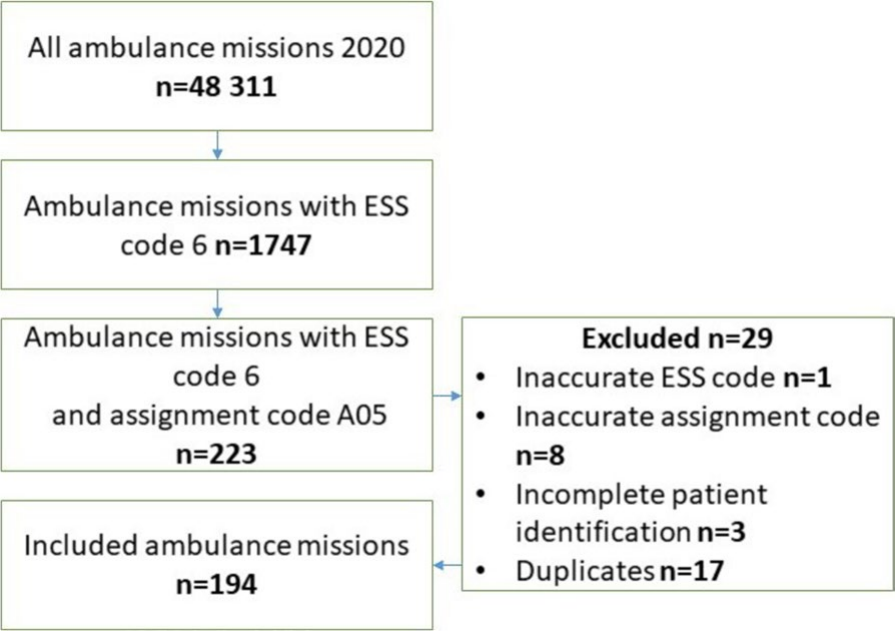
Flow chart showing the inclusion process of medical records for review

Data were abstracted from prehospital and hospital medical records. In all, there were 48311primary ambulance missions during the time of the survey. Among them did 1747 patients have ESS code six (abdominal pain) of which 223 patients had the assignment code A05 ‘triaged to self-care’. A total of 194 patients had no exclusion criteria and were therefor included in the study (Fig. 2).

The inclusion criteria included the following:

- All patients were assessed by ambulance during the time period from 1 January to 31 December, 2020, with EES code six and assignment code A05

The exclusion criteria included the following:

- The ambulance mission was categorised as assisting another ambulance.
- Inaccurate ESS code
- Inaccurate assignment code
- Incomplete patient identification

### Data collection

A data collection protocol was developed and pilot-tested before the data search. The protocol comprised data on on-scene assessments according to first and second surveys, measurements of vital parameters (executed and values), focused assessment, triage colour, contact with physician, use of triage handbook, written information to the patient and time on scene. These data were searched for in the EMS organisation’s prehospital medical record system (Ambulink). After conducting individual reviews in Ambulink, the respective prehospital medical records were followed up in the hospital’s medical record system (Melior), from which data on return visit to ED within 96 hours, hospitalisation, care time and ICD code were sampled.

Two of the authors (EO and PH) performed the data collection. They manually screened the medical records together. Both free text and fixed data were used. The relevant data were then transferred to a database where the data was de-anonymised and personal data linked to serial numbers were transferred to a separate codebook stored apart from the dataset. No inter-rater reliability test was performed, as the data collection was executed by two persons together.

### Data analysis

The outcome data were summarised using descriptive statistics. A univariate logistical regression analysis was used for predictors of return visits within 96 hours on binary (sex) and continuous variables (age and vital parameters). Significant variables in the univariate regression were considered eligible for inclusion in the multivariate model. A *P-*value of ≤0.05 was considered significant in the regression. All analyses were performed using SPSS 21.0 (SPSS Inc., Chicago, IL).

### Ethical issues

The study was accepted by the research ethics board of Stockholm, Sweden (Dnr 2021–03440) and conducted in agreement with the ethical references of the Swedish Research Council [17].

## Results

Amongst patients with abdominal pain who were triaged to self-care, 109 (55.6%) were females. The mean age of the patients was 56.3 years. On average, EMS teams spent a total of 28.0 minutes on the scene with each patient. The patients had normal vital signs (mean) and only three patients were assigned a RETTS colour of orange or red (Table 1).

**Table 1:** Demographic data, vital signs and triage

Variables	(***n*** = 194) (%)
Female	109 (55.6)
Men	85 (43.4)
Age, mean (years) ± SD^a^	56.3 ± 25.2
**RETTS colour**	
No triage performed	40 (20.4)
Blue	17 (8.7)
Green	86 (43,9)
Yellow	49 (25.0)
Orange	2 (1.0)
Red	1 (0.5)
Time on scene, mean (minutes) ± SD^a^	28.0 ± 12.6
Vital parameters and test, mean ± SD^a^	
Respiration rate/minute	17.1 ± 2.6
Oxygen saturation (%)	98.0 ± 1.8
Pulse rate/minute	79.7 ± 11.5
Systolic blood pressure mm/Hg	136.3 ± 18.9
Diastolic blood pressure mm/Hg	78.2 ± 11.3
Glasgow coma scale	15.0 ± 0.0
Body temperature C°	36.9 ± 0.7
Serum glucose mmol/L	7.1 ± 1.9

^a^*SD* Standard deviation

### Prehospital assessment

Compliance to 21 mandatory examinations was at 60.7%, with the greatest compliance attributed to the measurement of vital signs. Three patients had RETTS colours of orange and red, both of which are beyond the recommended triage colour for self-care. Contact with physicians at the EDs and the use of triage handbook had low compliance rates of 28.1 and 12.2%, respectively (Table 2).

**Table 2:** Prehospital performance of mandatory examinations and actions in connection to triage to self-care amongst patients with abdominal pain (*n* = 194)

Examination / action	n (%)
**Vital parameters**	
Respiration rate	181 (93.3)
Oxygen saturation	184 (93.9)
Pulse rate	184 (93.9)
Blood pressure	178 (90.8)
Degree of consciousness	139 (70.9)
Body temperature	179 (91.3)
**Anamnesis**	
**S**igns and symptoms	187 (95.4)
**A**llergies	61 (31.1)
**M**edications	113 (57.7)
**P**ertinent past history	167 (85.2)
**L**ast oral intake	22 (11.2)
**E**vents leading to the injury or illness	42 (21.4)
**O**nset	89 (45.4)
**P**rovokes or Palliates	63 (32.1)
**Q**uality	133 (67.9)
**R**adiates	62 (31.6)
**S**everity	54 (27.6)
**T**ime	132 (67.3)
**Focused examinations**	
Serum glucose	35 (17.9)
Abdominal auscultation	54 (27.6)
Abdominal inspection and palpation	110 (56.1)
**Average number of 21 mandatory assessment**	**13 (60.7)**
No triage	40 (20.4)
Triage outside recommendation	3 (1.5)
**Actions**	
Contact with physician at ED	55 (28.1)
Use of triage handbook	24 (12.2)
Written information to patient	164 (84.2)

### Patient outcome

Return visits with the same symptoms within 96 hours were observed in 32 cases (16.3%), whilst 11 (5.6%) were hospitalised. Fourteen patients (7.1%) were transported to a hospital by ambulance and 18 (9.2%) by other transport modes (Table 3). There were 19 different diagnoses amongst the patients required to have return visits, and all corresponded to abdominal pain as a symptom (Table 4). Of 6 patients (3.1%) who were diagnosed, the assessment of prehospital triage to self-care was considered inappropriate. One of the patients, suffering from a ruptured abdominal aortic aneurysm, died within 2 hours after the EMS assessment. The remaining patients were hospitalised from 3 to 120 hours and received surgical interventions, antibiotic therapy or fluid therapy (Table 4).

**Table 3:** Outcomes on return visits to ED and hospitalisation within 96 hours after prehospital assessment (*n* = 194)

Return visit 96 hours/hospitalisation	***n*** = 32 (%)
Ambulance transport	14 (43.7)
Other transport	18 (56.2)
Return visit mean (hours) ± SD^a^	20.1 ± 26.4
Emergency department	21 (65.6)
Hospitalisation	11 (34.3)
Length of stay mean (hours) ± SD^a^	75.4 ± 43.7

^a^*SD* Standard deviation

**Table 4:** ICD codes amongst patients with return visits 96 hours/hospitalisation and outcomes amongst patients with an incorrect prehospital triage assessment

ICD code (***n*** = 19)	Patients (***n*** = 32)	Incorrect prehospital triage (***n*** = 6)
A02.0 - Salmonella enteritis	1	
A04.7 - Enterocolitis due to *Clostridium difficile*	1	
A09.9 - Gastroenteritis and colitis of unspecified origin	1	
I71.3 - Abdominal aortic aneurysm, ruptured	1	Return visit after 1 hour. Deceased at the ED.
J12.9 - Viral pneumonia, unspecified	1	
K29.7 - Gastritis, unspecified	1	
K35.2 - Acute appendicitis with generalised peritonitis	1	Return visit after 3 hours. Hospitalized for 120 hours. Assessment and treatment: CT abdomen, laparoscopic appendectomy, antibiotics.
K35.3 - Acute appendicitis with localised peritonitis	1	Return visit after 3 hours. Hospitalised for 12 hours. Assessment and treatment: CT abdomen, laparoscopic appendectomy, antibiotics.
K35.8 - Acute appendicitis, other and unspecified	2	
K59.0 - Constipation	1	
K80.0 - Calculus of gallbladder with acute cholecystitis	1	
K80.2 - Calculus of gallbladder without cholecystitis	1	
K81.0 - Acute cholecystitis	2	
K81.9 - Cholecystitis, unspecified	4	
K85.1 - Biliary acute pancreatitis	2	Both return visits after 3 hours. One hospitalised 20 hours and one for 110 hours. Assessment and for cholangiopancreatography, ultrasound, fluid therapy.
K85.9 - Acute pancreatitis, unspecified	1	Return visit after 28 hours. Hospitalised for 48 hours. Assessment and treatment: CT abdomen, antibiotics, fluid therapy.
R10.4 - Other and unspecified abdominal pain	5	
R14 - Flatulence and related conditions	1	
R17.0 - Hyperbilirubinaemia with mention of jaundice, not elsewhere classified	2	
Triage - Assessed by nurse at ED	2	

### Predictor for return visits

**Fig. 3:**
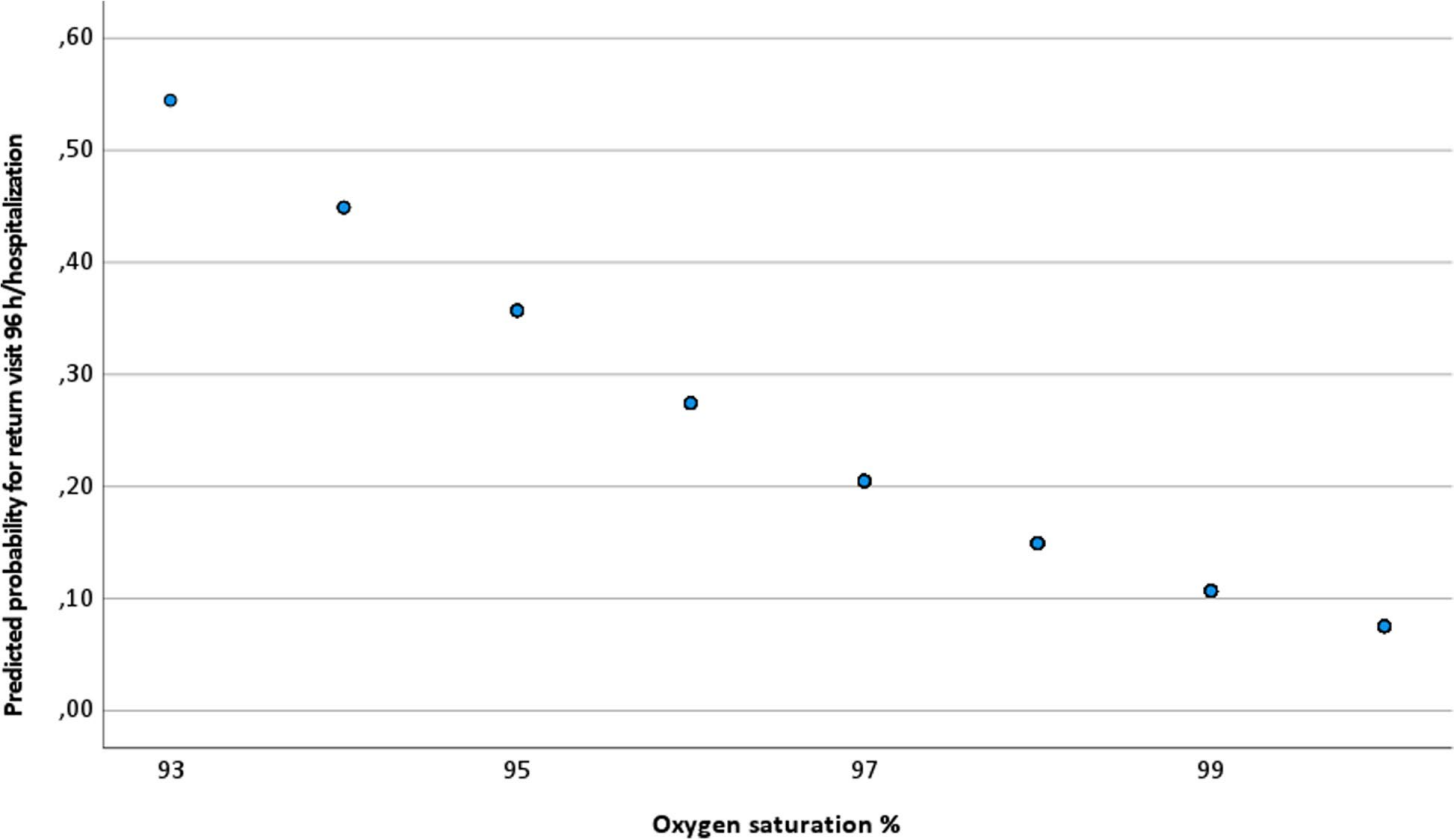
Predicted probability for return visits 96 hours/hospitalisation in relation to prehospital oxygen saturation

In the univariate logistic regression, there was only one significant variable that predicted outcome; thus, a multivariate logistic regression was not performed. A lower prehospital oxygen saturation level was a predictor for return visits, with an odds ratio of 0.68 (0.53–0.88) (Table 5). As shown in Fig. 3, the probability for return visits increased from 8% with a saturation of 100–55% to a saturation of 93%.

**Table 5:** Univariate predictors for return visits 96 hour/hospitalisation

Variable	Odds Ratio	95% CI	***P*** value
Age	1.01	0.99–1.02	0.27
Sex	1.15	0.53–2.49	0.72
Respiration rate	0.95	0.81–1.11	0.50
Oxygen saturation	0.68	0.53–0.88	< 0.01*
Systolic blood pressure	0.99	0.98–1.01	0.57
Pulse rate	1.00	0.97–1.03	0.82
Body temperature	1.54	0.78–3.01	0.21
Serum glucose	1.24	0.78–1.98	0.36

*Significant value

## Discussion

The results of this retrospective observational study confirm the hypothesis that, within the prehospital setting, it is challenging to safely assess patients with abdominal pain and triage them to self-care. The revisit rate was 16.3%. Furthermore, 6 patients (3.1%) were assessed to be incorrectly triaged to self-care by the EMS team, thereby endangering patient safety. This statement is made despite the fact that the EMS team’s compliance with guidelines can be considered relatively high.

The prehospital compliance to guidelines has been shown [18] to vary between 7.8–95%. Compliance with examination and treatment recommendations is generally lower compared to compliance with monitoring recommendations. This could also be observed in the present study. There are several reasons for the low guideline compliance in the prehospital setting, including the low evidence level in the guidelines that are being used, prevailing attitudes and workplace culture, and the physical format of the guidelines [18]. Accordingly, this can be an explanation for the poor use of the triage handbook in the present study. One study [19] has shown that paper-based guidelines can be cumbersome to use effectively in connection to patient assessment in the prehospital context.

The EMS team in this study had low compliance when it came to conducting focused examinations, such as abdominal auscultation, inspection and palpation. These are important examinations in the management of patients with abdominal pain. However, even properly performed examinations cannot rule out serious conditions. For example, one study showed that half of patients with peritonitis had normal bowel sounds and that palpation had low sensitivity and specificity for peritonitis [20]. In more than two-thirds of the cases examined, a physician was not contacted for advice; thus, there might be room for improvement to decide the right level of care. A previous study has reported decreased ambulance transport to the ED for patients with low priority conditions when ambulance nurses and physicians collaborate concerning the right level of care [21]. It is important to highlight that the consequences of inferior training in patient assessment already at the internship level of ambulance training. Incomplete assessments and anamnesis in the hospital field might be less dangerous when the patient is under observation for a longer time, the same shortcomings in patient assessment is more dangerous when “treat and release” in the prehospital setting.

The population in the current study is relatively young compared to patients with abdominal pain who are typically admitted to EDs [22]. This is a positive finding, as it probably means that most elderly patients are transported to hospitals. Elderly patients are more likely than younger patients to have severe aetiologies behind their abdominal pain. It has been shown that both mortality and misdiagnosis increase exponentially with each decade of age past 50 [20, 23].

Furthermore, EMS teams have limited opportunities to safely assess and examine patients with abdominal pain. For example, point-of-care blood tests are unusual, and it is impossible to have a patient undergo an X-ray examination on the spot. The common examination methods available are inspection, palpation and auscultation. It is also possible to measure ECG and vital parameters. Anamnesis is also a very important aspect of the examination done in the prehospital setting. At the same time, patients with abdominal pain are also difficult to assess at the ED, despite opportunities for more advanced examinations. Patient history and physical examinations have a sensitivity of 0.25 and a specificity of 0.92 compared to patient history, physical examination, laboratory analysis, acute abdominal series radiographs and non-enhanced helical computed tomography, with a sensitivity of 0.92 and a specificity of 0.90 [24]. Thus, overall, it is difficult to achieve a good assessment of patients with abdominal pain in a prehospital setting.

Meanwhile, we found that oxygen saturation is a critical clinical predictor associated with the risk of a return visit within 96 hours after the initial assessment, with a lower oxygen saturation indicating an increased risk of a return visit. The risk increased even above 90%. Previous studies have indicated that low oxygen saturation in the early phase is associated with an increased risk of adverse events amongst patients with acute myocardial infarction [25] and an increased risk of death amongst patients suffering from stroke [26]. The mechanisms behind these findings can only be speculated upon. A reasonable hypothesis is that when diseases in other organs are so severe that they influence respiration, it can be considered a serious sign.

Furthermore, our findings indicate the need for an accurate decision support tool so that not all patients have to be transported to an ED for evaluation. In developing such a tool, the definition of time-sensitive conditions must be carefully considered. Diseases, such as appendicitis and cholecystitis, should most likely be included in such a definition, regardless of any complication.

### Limitations

The main limitation of the present study is the low number of included patients. A study with a larger population could provide a better picture of the prehospital assessment and triage of patients with abdominal pain. Nevertheless, even though generalisability is limited by the small number of patients, the total number from 1 year of ambulance assignments is relatively large enough to highlight potential medical risks amongst patients triaged to self-care. Transferability to other ambulance organisations with similar guidelines should be approached with some caution.

Another limitation is the retrospective study design, which does not allow subsequent analyses of patients’ conditions or diagnoses when they are asked to remain at home. It has been suggested that there is a lack of a clear definition of what constitutes time-sensitive conditions [11]. The current study found that certain diseases could be included in further discussions to improve the precision of prehospital assessment and avoid delayed treatments for patients who require acute hospital care.

## Conclusion

Amongst the 1747 patients assessed by EMS due to abdominal pain, 223 (12.8%) were triaged to self-care of which 194 (87%) did not have any exclusion criteria. Among them 16.3% required return visits to EDs within 96 hours and 5.6% were hospitalised. Six patients had obvious time-sensitive conditions. Our study highlights the difficulties involved in the early assessment of patients with abdominal pain and the requirement for an accurate decision support tool.

## Data Availability

The datasets generated and/or analysed during the current study are not publicly available due the dataset is written in Swedish and needs to be translated to be useful for non-Swedish speakers, but are available from the corresponding author on reasonable request.

## Abbreviations

ED: Emergency Department
EMS: Emergency Medical Service
RETTS: Rapid Emergency Triage and Treatment System
VP: Vital Parameters
ESS codes: Emergency Signs and Symptoms
ECG: Electrocardiogram
AMLS: Advanced Medical Life Support
ABCDE: Airway, Breathing, Circulation, Disability and Exposure
